# Combining Machine Learning Models and Screening to Enhance Suicide Risk Identification for American Indian Patients: Retrospective Cohort Study

**DOI:** 10.2196/82669

**Published:** 2026-05-11

**Authors:** Novalene Alsenay Goklish, Emily E Haroz, Rohan R Dayal, Valentín Q Sierra, Roy Adams, Francene Larzelere Sinquah, Paul Rebman, Jacob L Taylor

**Affiliations:** 1 Department of International Health, Center for Indigenous Health, Johns Hopkins Bloomberg School of Public Health, 415 N. Washington St, Baltimore, MD, 21205, United States, 1 410-955-0011; 2 Department of Psychiatry and Behavioral Sciences, Johns Hopkins School of Medicine, Baltimore, MD, United States; 3 Department of Mental Health, Johns Hopkins Bloomberg School of Public Health, Baltimore, MD, United States

**Keywords:** suicide prevention, machine learning, American Indian and Alaska Native, screening, electronic health records, predictive modeling, suicide risk identification

## Abstract

**Background:**

American Indian and Alaska Native communities experience disproportionately high suicide rates. While machine learning (ML) models leveraging electronic health records have emerged as promising tools for suicide risk identification, the optimal integration of these models with existing screening practices remains unclear.

**Objective:**

The objective of this study was to compare parallel and serial testing strategies that combine an ML suicide risk model and the Ask Suicide-Screening Questions (ASQ) against using the ASQ alone. To achieve this, we conducted a retrospective secondary analysis of electronic health record data. The cohort consisted of adult emergency department visits at an Indian Health Service facility between October 1, 2019, and October 2, 2021.

**Methods:**

Sensitivity, specificity, predictive values, and 95% CIs were averaged across 10 cross-validated patient-level folds. The final sample included 7897 American Indian patients with 26,896 visits, 824 (3.1%) of which had a positive ASQ result and 102 (0.4%) of which had the outcome of suicide attempt or death within 90 days of the visit. The logistic regression ML model previously developed using Indian Health Service–specific data was operationalized at the 95th and 75th percentiles to evaluate high-risk and medium-risk thresholds, respectively. A sensitivity analysis was performed to evaluate identification approaches across all emergency department visits during this period.

**Results:**

The ML medium-risk threshold alone identified the most true positives (sensitivity: 0.782, 95% CI 0.648-0.915; specificity: 0.751, 95% CI 0.725-0.777; positive predictive value [PPV]: 0.012, 95% CI 0.009-0.014; negative predictive value [NPV]: 0.999, 95% CI 0.998-0.999) in comparison to the ML high-risk threshold alone (sensitivity: 0.429, 95% CI 0.287-0.572; specificity: 0.955, 95% CI 0.948-0.961; PPV: 0.035, 95% CI 0.022-0.048; NPV: 0.998, 95% CI 0.997-0.999) or the ASQ alone (sensitivity: 0.178, 95% CI 0.073-0.282; specificity: 0.970, 95% CI 0.968-0.971; PPV: 0.022, 95% CI 0.010-0.034; NPV: 0.997, 95% CI 0.996-0.998). Combining the ML high-risk threshold with the ASQ in series yielded the greatest positive predictive ability (PPV: 0.050, 95% CI 0.014-0.086) at the cost of reduced sensitivity (0.129, 95% CI 0.036-0.221). Finally, the parallel testing approach using the ML medium-risk threshold yielded the greatest sensitivity (0.795, 95% CI 0.671-0.920; specificity: 0.742, 95% CI 0.716-0.767; PPV: 0.012, 95% CI 0.009-0.014; NPV: 0.999, 95% CI 0.998-0.999) without missing any cases identified through screening.

**Conclusions:**

Unlike existing studies that evaluate ML and screening tools in isolation, this study innovates by assessing combined parallel and serial testing strategies in a real-world setting. We demonstrated that, while serial testing maximizes predictive accuracy, it is often infeasible. Instead, parallel testing brings value as a clinical “safety net” to catch at-risk patients missed by standard practices. Ultimately, integrating ML in suicide prevention requires balancing statistical accuracy with setting-specific, real-world workflows.

## Introduction

For the past 20 years, suicide rates in the United States have been steadily climbing. This is particularly true for American Indian and Alaska Native individuals, who face the highest rates of any racial or ethnic group and have experienced the steepest increases. In 2023, the US age-adjusted suicide rate was 14.1 per 100,000. However, the rate among American Indian and Alaska Native individuals was 23.8 per 100,000, representing a 44.2% increase since 2011 [1]. The burden of suicide in American Indian and Alaska Native populations reflects the confluence of historical, cultural, social, and economic factors that perpetuate intergenerational trauma and disparities in mental health resources. The devastating consequences of suicide on individuals, families, and communities underscore the urgent need for effective prevention strategies tailored to these populations.

Identifying individuals at risk of suicide remains a critical challenge in prevention efforts. Screening for suicide risk is now widely recommended as a critical strategy. Given the fact that most people who die by suicide saw a health care provider in the previous year [2], screening people at health care visits is designed to catch people before they engage in suicidal behaviors [3]. Screening can identify at-risk individuals who can then be connected with appropriate interventions, crisis services, or enhanced monitoring and support. Given this, the Joint Commission established National Patient Safety Goal 15.01.01, which requires accredited hospitals and behavioral health organizations to screen at-risk patients using evidence-based tools [4]. Others in the field of suicide prevention have argued for screening regardless of patient risk factors (universal screening) [5] with the notion that asking people about their past or current suicidal thoughts and behaviors does not confer risk and can provide an avenue for disclosure that may otherwise not be available. However, at the current time, the US Preventive Services Task Force has noted insufficient evidence to establish the benefits of universal suicide risk screening [6].

In parallel to advancements in screening, machine learning (ML) models using electronic health record (EHR) data have emerged as promising tools for suicide risk identification. Several large health systems, including the Veterans Health Administration [7], Kaiser Permanente [8], and academic medical centers [9], have developed and implemented EHR-based models that combine large numbers of patient variables to identify patterns and risk factors. These models have demonstrated promise, but not without their share of critiques as well. While predictive models perform generally comparably to existing screening tools [10,11], others have argued that they still suffer from too high a false-positive rate to engender trust and that full assessment by a trained clinician is the only way to ensure appropriate care [12]. However, clinicians have also been notably inaccurate [13-15], and their availability is limited in many places. Ultimately, the validity of predictive models depends critically on the quality and relevance of the data from which they are developed, and regardless of the risk identification strategy, implementation considerations are key to ensure seamless integration with existing care structures and workflows.

Despite the burden of suicide in American Indian and Alaska Native communities, there are few validated suicide risk identification tools and empirically supported interventions specifically designed and tested in American Indian and Alaska Native communities. Our research group has worked to address this gap through partnership with tribes in the US southwest and the Indian Health Service (IHS). Previously, we tested the performance of existing ML models developed by the Mental Health Research Network (MHRN) and Vanderbilt University in a majority American Indian population. The MHRN primary care model demonstrated good discrimination (area under the receiver operating characteristic curve [AUROC]=0.81) and outperformed both the Vanderbilt model and an augmented screening indicator (ie, Ask Suicide-Screening Questions [ASQ] acute positive or nonacute positive screen, a positive suicide risk assessment on the Columbia-Suicide Severity Rating Scale, 90-day history of ideation, or 5-year history of attempt) [16]. Building on these findings, we developed a context-specific suicide risk model using logistic regression and random forest approaches. These models, trained on EHR data, both achieved an AUROC of 0.83 for predicting suicide attempts or deaths within 90 days and again performed better than an augmented version of current practice [17].

However, a critical question remains in the context of implementation of screening tools: how should ML models be integrated with existing screening practices in clinical workflows? The optimal combination of these models and screening is unclear [18]. Several recent studies have begun to explore this question in other populations. Wilimitis et al [19] evaluated the integration of face-to-face screening with real-time ML in an adult primary care setting, finding that combined approaches improved risk identification. Aseltine et al [20] compared screening and risk algorithms in a pediatric emergency department (ED), demonstrating that the algorithms achieved higher sensitivity than screening when identifying the same proportion of patients as at risk. However, no studies have examined combined approaches specifically, and particularly not in American Indian and Alaska Native health settings.

This study addresses this gap by evaluating parallel and serial testing strategies that combine an IHS-specific ML model with the ASQ screening tool. We examined these approaches in ED visits. We aimed to (1) evaluate parallel testing strategies combining both approaches and (2) assess serial testing strategies that use ML to guide screening decisions. On the basis of the ML model’s ability to leverage longitudinal EHR data and prior studies showing that algorithms can add value to screening, we hypothesized that the ML model would identify more patients at risk than screening alone. We expected that parallel approaches would maximize case detection by capturing individuals identified through either method, whereas serial approaches would reduce false positives by requiring agreement between both tools, although potentially at the cost of missing some at-risk individuals. Our ultimate goal was to inform how the implementation of these tools can enhance suicide risk identification while being feasible within existing clinical workflows.

## Methods

### Overview

This study was a retrospective analysis conducted to evaluate the integration of an ML suicide risk model with suicide risk screening in an ED setting. The analysis was conducted as part of the Native-RISE (Risk Identification for Suicide and Enhanced Care) project, a multiyear partnership with several tribes in the US southwest. Building on prior work developing and validating ML models [4,5], we examined how parallel and serial testing strategies combining the model with the ASQ tool perform in identifying patients at risk of suicide attempts or deaths within 90 days of ED visits.

### Study Setting and Population

This study included 1 IHS service unit. The facility serves a geographically defined population of approximately 17,000 individuals and provides comprehensive health care services including emergency, outpatient, inpatient, and behavioral health care. The study population included all patients aged 18 years or older who presented to the ED between October 1, 2019, and October 2, 2021, and had a documented ASQ screening result. During this period, universal suicide risk screening using the ASQ was implemented in the ED. Patients were excluded if they did not have at least 90 days of follow-up time after their visit or if the visit type was not relevant for point-of-care suicide risk identification (eg, visits solely for vaccine administration or administrative record updates). All demographic characteristics, including race and ethnicity, were obtained from the EHR.

### The ML Model

The ML model used in this study was developed specifically for use in IHS settings and has been described in detail elsewhere [17]. Briefly, the model was developed using data from all adult patients (≥18 years) with visits between January 1, 2017, and October 2, 2021, at the same IHS facility (outcome data were included until December 31, 2021). Two modeling approaches were tested—logistic regression and random forest—both of which achieved comparable performance (AUROC=0.83). For this analysis, we used the logistic regression model given its greater interpretability.

Model features included demographics (age, sex, and race and ethnicity), clinical diagnoses (mental health conditions, substance use disorders, and chronic medical conditions), medications (psychotropic medications and pain medications), health care use patterns (visit frequency, ED visits, and hospitalizations), and prior suicide-related encounters (screening results, documented suicidal ideation, and prior attempts). Features were constructed using look-back periods ranging from 30 days to 5 years prior to each visit. Diagnosis codes were identified using *International Classification of Diseases, 10th Revision*, codes and established code lists from the MHRN.

The model generates a predicted probability of suicide attempt or death within 90 days following each ED visit. For this analysis, we examined model performance at 2 risk thresholds: the 95th percentile (high risk) and the 75th percentile (medium or high risk). These thresholds were selected to balance sensitivity and positive predictive value (PPV) while maintaining clinical feasibility.

### The ASQ Tool

The ASQ is a brief, 4-item screening tool developed by the National Institute of Mental Health for use in medical settings. Patients are asked about (1) wishing to die in the previous few weeks, (2) feeling that they would be better off dead in the previous few weeks, (3) thoughts about killing themselves in the previous week, and (4) any previous suicide attempts [21]. Any affirmative response results in a fifth acuity question that assesses current suicidal ideation—positive responses result in an acute positive classification, and those without current suicidal ideation are classified as nonacute positive. For our analysis, we combined nonacute positive and acute positive responses into 1 category of positive ASQ screening results to estimate summary statistics. In our setting, patients screened as acute or nonacute positive receive additional risk assessment by behavioral health staff.

The ASQ has demonstrated validity in pediatric EDs and medical settings, with high concordance with more extensive assessments [22-24]. However, the ASQ has never been validated in American Indian and Alaska Native populations. Implementation challenges can include when and how the tool is administered and completion rates. In our study setting, the ASQ was either completed on paper or administered verbally. Documented completion during the study period was 62.7% (26,896/42,915).

### Testing Strategies

In addition to estimating the impact of the ASQ or ML approaches alone, we evaluated 2 approaches for combining ML and screening, described in the following sections.

#### Parallel Testing

**Figure 1.**
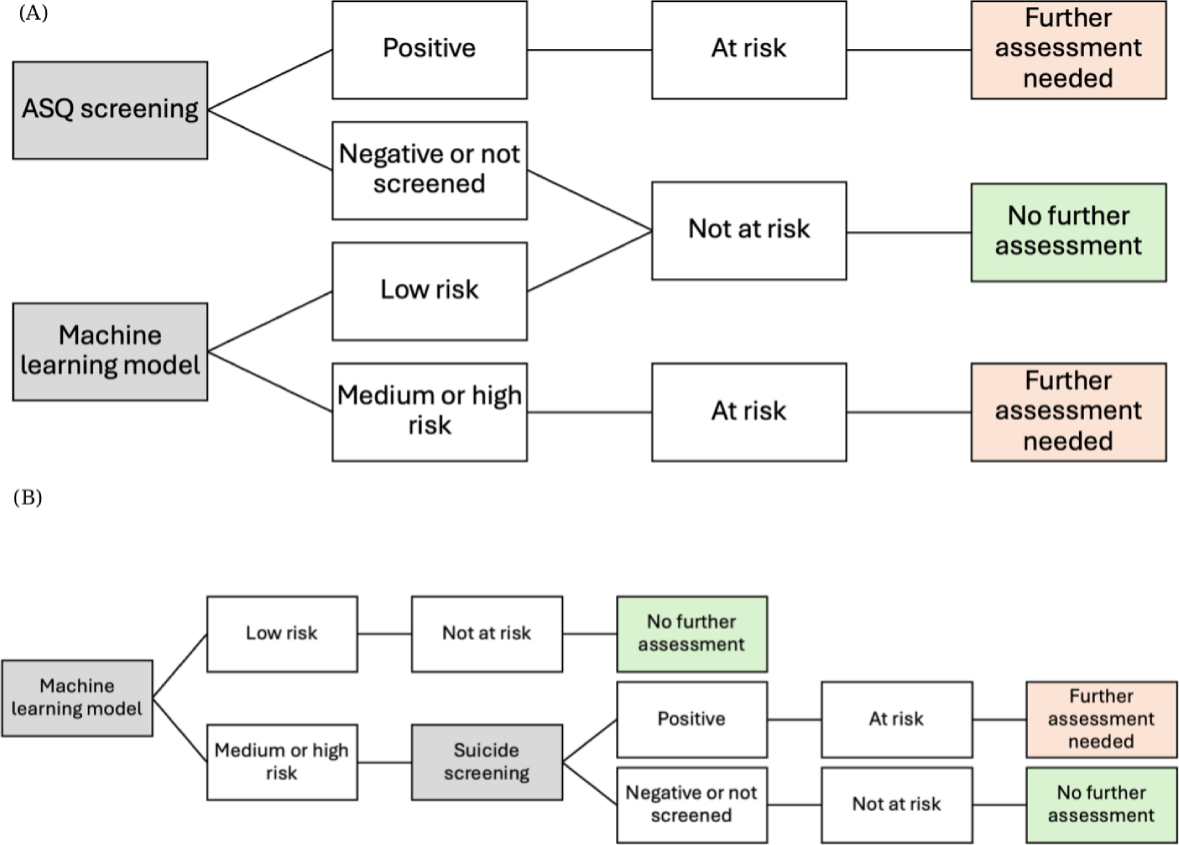
(A) Pragmatic approach to parallel testing: the machine learning model is used to verify negative screens or assist when screening was negative or did not occur. (B) A hypothetical approach: this approach may be optimal because the machine learning model has higher sensitivity and moderate specificity and is noninvasive and easier to administer. ASQ: Ask Suicide-Screening Questions.

Patients were classified as at risk if they were screened as positive on the ASQ *or* if the ML model classified them as medium or high risk (≥75th percentile) or high risk (≥95th percentile). This approach uses both tools simultaneously, with either tool being sufficient to flag risk. Figure 1A shows a parallel approach in which the ML model verifies negative screens or assists when screening is missed.

#### Serial Testing

Patients were classified as at risk if the ML model classified them as medium or high risk (≥75th percentile) or high risk (≥95th percentile) *and* they were screened as positive on the ASQ. This approach uses the ML model as a first-stage filter, with screening administered only to those flagged by the model. Figure 1B shows this hypothetical approach.

### Outcomes

The primary outcome was suicide attempt or death by suicide within 90 days following the ED visit. Suicide attempts were identified using *International Classification of Diseases, 10th Revision*, diagnosis codes for intentional self-harm [17]. Suicide deaths were identified through the Tribe’s community-based surveillance system, which maintains comprehensive mortality records including cause of death determinations [25]. Data were right censored on December 31, 2021, to allow for complete 90-day follow-up periods. Serial and parallel testing approaches were coded empirically using the medium- and high-risk thresholds for the ML model and ASQ results recorded in the EHR.

### Statistical Analysis

For each testing strategy, we assessed the sensitivity, specificity, PPV, and negative predictive value (NPV) with 95% CIs. Performance metrics were calculated averaging across the 10 cross-validation folds created at the patient level used during model development, with 95% CIs calculated for point estimates using cross-validated SE estimates across folds.

We compared performance across testing strategies descriptively given the rarity of the outcome events, focusing on the trade-offs between sensitivity and PPV. Given the low base rate of suicide attempts and deaths (102/26,896, 0.4% of visits), we paid particular attention to PPV as this metric is driven by underlying prevalence of the outcome, indicates the proportion of flagged patients who experience the outcome, and directly relates to resource allocation and clinical burden.

### Sensitivity Analysis

We conducted a sensitivity analysis replicating all analyses in the full sample of ED visits including those without documented ASQ screening (n=42,915 ED visits). This analysis provides insights into real-world performance, where screening completion rates may vary. Results are shown in Multimedia Appendix 1.

### Ethical Considerations

#### Human Subject Ethics Review, Exemptions, or Approvals

The study was reviewed and declared non–human subject research by the Institutional Review Board of Johns Hopkins Bloomberg School of Public Health (17333) and the Phoenix Area IHS Office Institutional Review Board (20.05). The Tribe approved the study through the Tribal Health Board and Tribal Council. The reporting of findings abides by STROBE (Strengthening the Reporting of Observational Studies in Epidemiology) guidelines [7].

#### Informed Consent

Informed consent was waived through a HIPAA (Health Insurance Portability and Accountability Act) authorization granted to the research team by the IHS Institutional Review Board for secondary data analysis of EHR data.

#### Privacy and Confidentiality

Research data were accessed through a data use agreement between the IHS and Johns Hopkins University. These data were protected during storage and analysis using a secure and HIPAA-compliant computing platform provided by the Johns Hopkins University School of Medicine. Research data were deidentified after preprocessing to ensure privacy and confidentiality. Patient-level statistics with substantially small sample sizes (n<5) were omitted to ensure that potentially identifying information was not included in this manuscript.

#### Compensation Details

Compensation for this study is not applicable as this was a secondary analysis of EHR data.

## Results

### Study Population

Between October 1, 2019, and October 2, 2021, there were 42,915 ED visits. Of these 42,915 visits, 26,896 (62.7%) had documented ASQ screening results and were included in the primary analysis. These visits represented 7897 unique patients, with a median of 2 (IQR 1-4) ED visits per patient. Patient demographics are presented in Table 1. The median age was 38 (IQR 28-54) years. Most patients were female (4241/7897, 53.7%) and identified as American Indian (7734/7897, 97.9%). Of the 7897 patients, 614 (7.8%) were screened as positive on the ASQ at one or more of their ED visits during the study period.

**Table 1.** Patient and visit characteristics (n=7897).

Characteristics	Values
Age (y), median (IQR)	38 (28-54)
Female, n (%)	4241 (53.7)
Race or ethnicity, n (%)	
American Indian	7734 (97.9)
Other or unknown	163 (2.1)
Any positive ASQ^a^ screen, n (%)	614 (7.8)
ED^b^ visits (n=26,896)	
Visits per patient, median (IQR)	2 (1-4)
Positive ASQ screen, n (%)	824 (3.1)

a ASQ: Ask Suicide-Screening Questions.

b ED: emergency department.

### Outcomes

Suicide attempts or deaths within 90 days of an ED visit occurred in 0.4% (102/26,896) of the visits among 0.7% (55/7734) of unique patients. Of the 55 patients who experienced an outcome, 14 (25.5%) had been screened as positive on the ASQ at least once in the 90 days prior to a suicide attempt or death, whereas 41 (74.5%) were never screened as positive on the ASQ at an ED visit within 90 days of a suicide attempt. No one who died by suicide was screened as positive on the ASQ at any visit within 90 days of their death.

### Performance of Individual Approaches

Table 2 shows the sensitivity, specificity, PPV, and NPV for each testing strategy. The ASQ screening alone exhibited a sensitivity of 0.178 (95% CI 0.073-0.282), specificity of 0.970 (95% CI 0.968-0.971), PPV of 0.022 (95% CI 0.010-0.034), and NPV of 0.997 (95% CI 0.996-0.998).

The ML model alone using the 75th percentile threshold (medium or high risk) achieved a sensitivity of 0.782 (95% CI 0.648-0.915), specificity of 0.751 (95% CI 0.725-0.777), PPV of 0.012 (95% CI 0.009-0.014), and NPV of 0.999 (95% CI 0.998-0.999). At the more stringent 95th percentile threshold (high risk only), the model achieved a sensitivity of 0.429 (95% CI 0.287-0.572), specificity of 0.955 (95% CI 0.948-0.961), PPV of 0.035 (95% CI 0.022-0.048), and NPV of 0.998 (95% CI 0.997-0.999). The ML model outperformed ASQ screening on sensitivity and NPV at the evaluated thresholds; however, because the model included prior screening and ideation features, this comparison reflects the incremental value of longitudinal EHR information rather than a contemporaneous head-to-head assessment of the index screening alone.

**Table 2.** Parallel and serial testing of machine learning (ML) model risk categories and suicide screening among visits with an administered suicide screening survey (n=7897 patients; n=26,896 emergency department visits).

	Sensitivity (95% CI)	Specificity (95% CI)	PPV^a^ (95% CI)	NPV^b^ (95% CI)
Classification alone				
ML model high risk (≥95th percentile)	0.429 (0.287-0.572)	0.955 (0.948-0.961)	0.035 (0.022-0.048)	0.998 (0.997-0.999)
ML model medium or high risk (≥75th percentile)	0.782 (0.648-0.915)	0.751 (0.725-0.777)	0.012 (0.009-0.014)	0.999 (0.998-0.999)
ASQ^c^	0.178 (0.073-0.282)	0.970 (0.968-0.971)	0.022 (0.010-0.034)	0.997 (0.996-0.998)
Parallel				
ASQ or ML model high risk (≥95th percentile)	0.478 (0.344-0.612)	0.934 (0.928-0.940)	0.027 (0.018-0.036)	0.998 (0.997-0.999)
ASQ or ML model medium or high risk (≥75th percentile)	0.795 (0.671-0.920)	0.742 (0.716-0.767)	0.012 (0.009-0.014)	0.999 (0.998-0.999)
Serial				
ML model high risk AND ASQ	0.129 (0.036-0.221)	0.991 (0.989-0.993)	0.050 (0.014-0.086)	0.997 (0.996-0.998)
ML model medium or high risk AND ASQ	0.164 (0.059-0.268)	0.980 (0.978-0.981)	0.029 (0.012-0.045)	0.997 (0.996-0.998)

a PPV: positive predictive value.

b NPV: negative predictive value.

c ASQ: Ask Suicide-Screening Questions.

### Performance of Combined Approaches

Parallel testing strategies, where patients were flagged if they were screened as positive *or* were classified as at risk by the ML model, substantially improved sensitivity compared to screening alone. When combining ASQ with the ML model at the 95th percentile, sensitivity increased to 0.478 (95% CI 0.344-0.612), with a specificity of 0.934 (95% CI 0.928-0.940), PPV of 0.027 (95% CI 0.018-0.036), and NPV of 0.998 (95% CI 0.997-0.999). This approach identified approximately half of patients who subsequently experienced an outcome without missing any patients who would have been caught through screening alone.

When combining the ASQ with the ML model at the 75th percentile in parallel, sensitivity further improved to 0.795 (95% CI 0.671-0.920), with a specificity of 0.742 (95% CI 0.716-0.767), PPV of 0.012 (95% CI 0.009-0.014), and NPV of 0.999 (95% CI 0.998-0.999). This more inclusive parallel approach identified 4 out of 5 patients who experienced an outcome but resulted in substantially more potential false positives.

Serial testing strategies, where patients were flagged only if *both* the ML model classified them as at risk *and* they were screened as positive, substantially improved PPV compared to other approaches. Using the 95th percentile threshold, the serial approach achieved a sensitivity of 0.129 (95% CI 0.036-0.221), specificity of 0.991 (95% CI 0.989-0.993), PPV of 0.050 (95% CI 0.014-0.086), and NPV of 0.997 (95% CI 0.996-0.998). This represents more than a 2-fold improvement in PPV compared to ASQ screening alone (0.050 vs 0.022), although at the cost of reduced sensitivity.

Using the 75th percentile threshold, the serial approach achieved a sensitivity of 0.164 (95% CI 0.059-0.268), specificity of 0.980 (95% CI 0.978-0.981), PPV of 0.029 (95% CI 0.012-0.045), and NPV of 0.997 (95% CI 0.996-0.998). This approach still provided a 32% increase in PPV compared to screening alone (0.029 vs 0.022) with slightly improved specificity. The CIs for the serial testing approaches in both the 95th and 75th percentile thresholds were notably wide, particularly for sensitivity (eg, 0.036-0.221 and 0.059-0.268, respectively). This reflects the small number of true positive cases identified through these highly specific strategies.

### Sensitivity Analysis

Results from the sensitivity analysis including all ED visits (n=42,915) regardless of screening completion are presented in Multimedia Appendix 1. Performance patterns were similar to those of the primary analysis, although with slightly attenuated sensitivity across all approaches due to the larger denominator of missed screening opportunities.

## Discussion

### Principal Results

We aimed to evaluate strategies for combining ML models with suicide risk screening in an ED that serves American Indian communities. Three key findings emerged. First, when operationalizing the ML model at a lower threshold, we substantially increased sensitivity over screening alone, identifying 78.2% of patients who subsequently attempted or died by suicide compared to 17.8% of patients via screening alone. Second, parallel testing combining both approaches improved sensitivity to 0.795 while maintaining reasonable specificity (0.742). Third, serial testing requiring both positive ML classification and positive screening achieved more than a 2-fold improvement in PPV (0.050 vs 0.022) but with reduced sensitivity (0.129 vs 0.178).

What constitutes adequate performance for suicide risk identification depends on the intended use, available resources, and tolerance for missed cases vs false alarms [12,26]. Our findings are consistent with those of recent meta-analyses showing pooled sensitivities of 45% to 82% and PPVs of 6% to 17% for ML algorithms for suicide risk identification [12]. The PPVs we observed (0.012-0.050) reflect the mathematical reality that low prevalence constrains PPV even when sensitivity and specificity are reasonable, a challenge inherent to predicting any rare outcome.

### Comparison With Prior Work

Our results extend our prior work demonstrating that IHS-specific ML models (AUROC=0.83) outperform screening-based approaches [17] and parallel recent pediatric ED findings by Aseltine et al [20] showing that risk algorithms correctly identified 51% of subsequent attempts compared to 37% through screening. The consistency across populations suggests that ML’s added value may be generalizable.

Critically, neither the ASQ nor other suicide screening instruments have been validated in American Indian and Alaska Native populations [27,28]. Our findings, showing a sensitivity of only 0.178, suggest that the tool may perform differently in this population or that implementation challenges limit effectiveness. The ASQ has shown much stronger predictive accuracy in non–American Indian and Alaska Native pediatric populations [24,29] but has not been validated against attempts or deaths in adult populations. Implementation challenges can include when and how questions are asked, cultural differences in communication patterns, or trust in health care systems [30,31]. While we wait on specific validation data, this issue reflects a broader pattern of concern: tools developed with majority populations are applied to high-burden minority populations without proper validation, risking perpetuation of health inequities [32].

Serial testing substantially improved PPV but may be infeasible given that universal screening is already implemented in many settings [33]. Ethically, withholding screening from ML-identified low-risk patients is problematic, particularly if they subsequently disclose suicidality. As Wilimitis et al [19] noted, approaches that rely primarily on ML to dictate workflows face significant ethical obstacles and logistical challenges when patients disclose risk factors. ML approaches also often depend on a patient having a recent history with the health system, creating a challenge to prediction when patients are new or have not been seen in recent years.

This creates a tension: the strategy with the best PPV is impractical in cases in which universal screening is already established or in settings when past history with the health system is not as well established. One pragmatic solution (Figure 1) is using ML to verify negative screens or flag high-risk patients when screening is missed. In our data, 37.3% (16,019/42,915) of ED visits lacked documented screening, and others were false negatives. For these cases, ML could serve as a safety net. For patients who are screened as negative but are classified as high risk by ML, clinicians could be alerted to reassess. This preserves universal screening while leveraging ML to reduce false negatives.

High false-positive rates raise concerns about resource strain and patient harm. However, 3 considerations provide context. First, many flagged patients who do not go on to attempt suicide may benefit from intervention as health care providers may use algorithm features to identify and intervene in other underlying conditions through connection with services or treatment. Second, false positives must be weighed against the consequences of false negatives in communities with starkly elevated suicide rates. Third, acceptability depends on the intervention burden; brief safety assessments and follow-up contacts may justify higher false-positive rates than intensive interventions such as involuntary hospitalization. Notably, widely accepted screening programs such as mammography have comparable PPVs (approximately 4% on the lower end) [34], although breast cancer screening has decades of mortality reduction evidence that suicide screening currently lacks [35].

### Limitations

The findings are specific to a single tribal community, limiting generalizability. EHR-based outcome identification may miss undocumented attempts, potentially underestimating risk differentially across patient groups. We analyzed only visits with documented screening (26,896/42,915, 62.7% of total ED visits), which may not reflect real-world performance. We did not collect standardized ASQ implementation fidelity measures (mode, timing, staff training, privacy, or cultural adaptation), which may have influenced observed sensitivity. The ASQ currently lacks validation in American Indian and Alaska Native populations [27,28], and implementation challenges may limit its effectiveness. While the model was developed and tested in the same facility, it represents a retraining of an existing model from a completely different population [17], so while external validation for our metrics is warranted, the transportability of the underlying ML model has considerable strengths.

An additional important limitation is that the ML model depends on longitudinal EHR data to generate predictions [17]. Patients with limited or no prior health care encounters—such as those new to the system or seeking care for the first time—would have minimal data for the algorithm to analyze. While this is less common in our study setting given the reservation-based health care system with limited alternatives, it remains a significant consideration. This dependence on prior health care engagement highlights a fundamental value of screening tools: they assess current suicide risk through self-report regardless of prior history. This represents another reason why ML cannot replace screening, particularly for patients with sparse use patterns, and supports the rationale for combined rather than stand-alone approaches.

### Conclusions

Unlike existing research that primarily evaluates ML models and screening tools in isolation, this study innovates by evaluating combined parallel and serial testing strategies within a real-world American Indian ED setting. We demonstrate that, while ML models substantially outperform suicide risk screening alone, combining approaches yields distinct trade-offs. Serial testing improves PPV, but it remains practically and ethically infeasible in universal screening settings, either due to the ethical barriers of withholding treatment from individuals who disclose risk on the ASQ or because ML methods may require a history of several visits to adequately classify risk. Instead, parallel testing brings immediate value to the field by preserving universal screening and allowing ML to act as a clinical “safety net” to identify at-risk patients who are missed by current practices. Critically, these findings highlight that the most statistically accurate identification approach is not always practical. The optimal real-world integration of ML risk prediction requires careful consideration of setting-specific workflows to ensure that these approaches do not inadvertently perpetuate resource constraints or health inequities. Future work should focus on prospective evaluation of clinical outcomes, external validation in diverse settings, and continued community-engaged development to ensure that tools meet the needs of American Indian and Alaska Native communities facing the highest suicide burden.

## Supplementary material

10.2196/82669 Multimedia Appendix 1 Parallel and serial testing of machine learning model risk categories and suicide screening in the full sample (n=9224 patients; n=42,915 emergency department visits).

## Abbreviations

ASQ: Ask Suicide-Screening Questions
AUROC: area under the receiver operating characteristic curve
ED: emergency department
EHR: electronic health record
HIPAA: Health Insurance Portability and Accountability Act
IHS: Indian Health Service
MHRN: Mental Health Research Network
ML: machine learning
NPV: negative predictive value
PPV: positive predictive value
STROBE: Strengthening the Reporting of Observational Studies in Epidemiology
