# Supplementary material for: Combining Machine Learning Models and Screening to Enhance Suicide Risk Identification for American Indian Patients: Retrospective Cohort Study
Source: J Med Internet Res. 2026 May 11;28:e82669. doi: 10.2196/82669 (PMC13160258; doi:10.2196/82669)
Supplement: Multimedia Appendix 1 [file jmir-v28-e82669-s001.docx]

| **Table 1.** Patient and Visit Characteristics | | |
| --- | --- | --- |
| **Patients** | ***N=*9,244** |  |
| Age in years, median (IQR) | 38 (28, 54) |  |
| Female, n (%) | 4,874 (52.7) |  |
| Race/Ethnicity, n (%) |  |  |
| American Indian | 8,975 (97.1) |  |
| Other or Unknown | 269 (2.9) |  |
| Any positive ASQ screen, n (%) | 614 (7.8) |  |
| **ED Visits** | ***N=*42,915** |  |
| Visits per patient, median (IQR) | 3 (1, 6) |  |
| Positive ASQ screen, n (%) | 824 (1.9) |  |

| **Table 2 –** *Parallel and serial testing of machine learning model risk categories and suicide screening among full sample*(*N=*9,224 Patients; 42,915 Visits) | | | | |
| --- | --- | --- | --- | --- |
|  | Sensitivity  (95% CI) | Specificity  (95% CI) | PPV  (95% CI) | NPV  (95% CI) |
| **Classification Alone** |  |  |  |  |
| ML model high risk  *(<95th percentile)* | 0.398  (0. 269, 0.527) | 0.950  (0.945, 0.956) | 0.037  (0.022, 0.052) | 0.997  (0.996, 0.998) |
| ML model medium or high risk  *(<75th percentile)* | 0.490  (0.336, 0.644) | 0.903  (0.892, 0.914) | 0.023  (0.015, 0.032) | 0.997  (0.997, 0.998) |
| ASQ | 0.089  (0.042, 0.136) | 0.981  (0.980, 0.982) | 0.022  (0.010, 0.034) | 0.996  (0.995, 0.996) |
| **Parallel** |  |  |  |  |
| ASQ **or** ML model high risk *(<95th percentile)* | 0.428  (0.301, 0.575) | 0.937  (0.932, 0.942) | 0.031  (0.020, 0.043) | 0.997  (0.996, 0.998) |
| ASQ **or** ML model medium or high Risk *(<75th percentile)* | 0.734  (0.707, 0.762) | 0.886  (0.876, 0.896) | 0.014  (0.011, 0.016) | 0.999  (0.998, 0.999) |
| **Serial** |  |  |  |  |
| ML model high risk **AND** ASQ | 0.059  (0.020, 0.098) | 0.994  (0.993, 0.995) | 0.050  (0.014, 0.086) | 0.996  (0.995, 0.996) |
| ML model medium or high risk **AND** ASQ | 0.079  (0.036, 0.123) | 0.987  (0.986, 0.988) | 0.029  (0.012, 0.045) | 0.996  (0.995, 0.996) |

Abbreviations: ASQ, Ask Suicide Questionnaire; CI, confidence interval; NPV, negative predictive value; PPV, positive predictive value; ML, machine learning.
